# Ceramide and Ischemia/Reperfusion Injury

**DOI:** 10.1155/2018/3646725

**Published:** 2018-01-21

**Authors:** Xingxuan He, Edward H. Schuchman

**Affiliations:** Department of Genetics & Genomic Sciences, Icahn School of Medicine at Mount Sinai, 1425 Madison Avenue, New York, NY 10029, USA

## Abstract

Ceramide, a bioactive membrane sphingolipid, functions as an important second messenger in apoptosis and cell signaling. In response to stresses, it may be generated by de novo synthesis, sphingomyelin hydrolysis, and/or recycling of complex sphingolipids. It is cleared from cells through the activity of ceramidases, phosphorylation to ceramide-1-phosphate, or resynthesis into more complex sphingolipids. Ischemia/reperfusion (IR) injury occurs when oxygen/nutrition is rapidly reintroduced into ischemic tissue, resulting in cell death and tissue damage, and is a major concern in diverse clinical settings, including organ resection and transplantation. Numerous reports show that ceramide levels are markedly elevated during IR. Mitochondria are major sites of reactive oxygen species (ROS) production and play a key role in IR-induced and ceramide-mediated cell death and tissue damage. During the development of IR injury, the initial response of ROS and TNF-alpha production activates two major ceramide generating pathways (sphingomyelin hydrolysis and de novo ceramide synthesis). The increased ceramide has broad effects depending on the IR phases, including both pro- and antiapoptotic effects. Therefore, strategies that reduce the levels of ceramide, for example, by modulation of ceramidase and/or sphingomyelinases activities, may represent novel and promising therapeutic approaches to prevent or treat IR injury in diverse clinical settings.

## 1. Introduction

**Figure 1:**
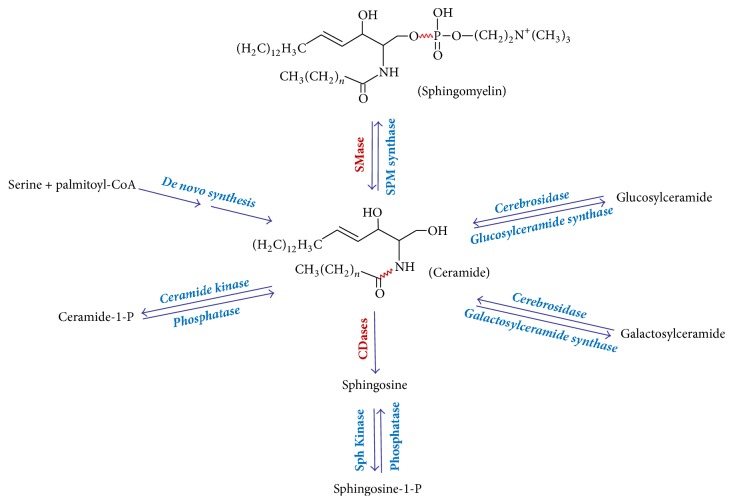
Scheme of ceramide metabolism.

Sphingolipids are essential structural components of all cell membranes and highly bioactive compounds that play important roles in signal transduction and numerous other cellular processes such as cell proliferation, differentiation, and apoptosis. Ceramide is a central component of sphingolipid structure and metabolism. There are several ways to generate ceramide in mammalian cells (Figure 1): hydrolysis of sphingomyelin, de novo synthesis from palmitoyl-CoA and serine, catabolism of glucosylceramide and galactosylceramide, synthesis from sphingosine and fatty acid, and dephosphorylation of ceramide-1-phosphate. However, these multiple pathways for ceramide generation do not contribute evenly, and there are many cell specific and other regulatory checkpoints that activate the specific pathways.

Over the past two decades, ceramide has been recognized as a key bioactive lipid and second messenger that mediates the proliferation, survival, and death of cells. Ceramide's role as a second messenger was first recognized in 1990 in the context of HL-60 cell proliferation [1]. In the late 1990s, further publications demonstrated the accumulation of ceramide in response to diverse cellular stresses, like infection, radiation, cytokines, death ligands, reactive oxygen species (ROS), and others [2–4]. Stress-induced ceramide accumulation leads to reorganization of the plasma membrane and formation of ceramide-rich platforms, often referred to as “rafts.” These raft platforms recruit and cluster death receptors and signaling molecules at the cell membrane to facilitate amplification of signal transduction cascades and activation of cell death signaling pathways [5–7]. Increasing evidence also reveals that ceramide elevation is involved in diverse diseases, like diabetes [8], cardiovascular disease [9, 10], Alzheimer's disease [11, 12], and others.

In addition, the role of ceramide in the pathogenesis of ischemia/reperfusion (IR) injury has attracted considerable attention. IR injury occurs when the blood supply returns to tissues after a period of ischemia or lack of oxygen, resulting in cell death and tissue damage. Although there are no standard classifications of ischemia and IR, to better understand IR injury it can be classified into different phases or types according to the time and extent of the insults. For example, total or partial ischemia is defined as full obstruction of the blood vessel or blockage of a small area only, respectively. Brief/early phase or prolonged/later phase ischemia is defined by the length of time the tissues lack oxygen, from minutes to hours, respectively. Reperfusion results in a series of pathological changes associated with the time and extent of the ischemia. Mild or severe ischemia and IR are defined based on a combination of the area of blockage and time following reperfusion. In organ transplantation events, ischemia also can be classified as cold or warm ischemia. Generally, cold ischemia (4–7°C) can be protective due to reduced metabolic processes and cellular ATP demand at lower temperature, whereas warm ischemia (37°C) is usually harmful to cells and molecular pathways [13].

The incidence of IR injury is substantial. There are millions of individuals each year in the US suffering from cardiac infarction, stroke, thrombosis, blood vessel clamped surgery, and organ failure requiring transplantation. Restoration of blood supply should protect the tissues from damage, but reperfusion often leads to injury. Even though it is widely accepted that this IR injury results from the production of ROS, recruiting neutrophils, macrophages, and inflammatory mediators to the injured tissue, the mechanisms of IR injury remain to be elucidated. In this review we will concentrate on the molecular mechanisms of ceramide elevation and tissue damage observed during IR injury.

The first report indicating the involvement of ceramide in IR injury was published by Bradham et al. in 1997 [14]. They demonstrated that there was a significant elevation of ceramide during liver transplantation (cold ischemia and warm reperfusion). In the same year, the accumulation of ceramide also was observed in both heart and renal IR injuries [15, 16]. After more than a decade of investigations, it has become clear that ceramide generation plays a key role during IR injury. A comprehensive understanding of the mechanism behind these changes has not yet been clarified, as several sphingolipid-metabolizing enzymes have been involved in ceramide generation during IR injury. A further understanding of these mechanisms could lead to more targeted therapies to prevent ceramide generation during IR.

## 2. The Sphingomyelin/Ceramide Signaling Pathway and IR

Two major pathways, sphingomyelin hydrolysis and de novo biosynthesis, have been implicated in the generation of ceramide. Both pathways may be activated separately or in parallel depending on stimuli or on the cell type [17]. Diverse oxidative stresses induce cell apoptosis or necrosis and tissue damage via activation of SMases, resulting in sphingomyelin hydrolysis with ceramide generation. The accumulation of ceramide has been reported in multiple models of ischemia, including rat cerebral cortex and gerbil hippocampus ischemia [18, 19], as well as in models of reperfusion injury, including artery occlusions in rat brain, liver, and heart [20–22]. In renal and cardiac IR injury models, activation of SMase and accumulation of ceramide were observed in the later phase of IR. Meanwhile, decreases in sphingomyelin corresponded to the increases in ceramide [23]. A few studies describe endogenous ceramide accumulation in brain via activation of a SMase leading to sphingomyelin hydrolysis during severe and lethal cerebral IR. In vitro, after hypoxia/reoxygenation of cardiac myocytes, the early responses (peaking at 10 min) included the activation of neutral SMase and low level ceramide accumulation [24]. Factor associated with neutral SMase activation (FAN), a protein that links neutral SMase to the tumor necrosis factor alpha (TNF*α*) receptor, mediates activation of neutral SMase and subsequent apoptosis. The expression of a dominant-negative FAN in rat cardiomyocytes almost completely abrogated hypoxia/reoxygenation-induced cell death, whereas overexpression of wild-type FAN led to an exacerbation of IR injury [25].

During ischemia, aerobic metabolism interrupts due to the lack of oxygen supply. Build-up of oxidative cell damage occurs during reperfusion to sites of ischemia, which is characterized by excess ROS generation and inflammatory cytokine recruitment [22, 26]. Several studies have shown that ceramide generation by SMase contributes to ROS and TNF*α* induced cell death and tissue damage [27–29]. For example, Wistar rats subjected to total liver ischemia followed by reperfusion had significant accumulation of TNF*α* and an increase of SMase activity that coincided with IR injury [30]. In an in vitro study, overexpression of acid ceramidase protected murine fibroblasts from TNF*α*-induced cell apoptosis by shifting elevated ceramide towards cell survival sphingosine-1-phosphate [31]. In TNF*α* gene knockout mice, IR-induced hepatic apoptosis was attenuated, and animal survival was prolonged compared to wild-type mice. These data have further identified TNF*α* as a critical mediator in hepatic IR injury [32]. Ceramide and TNF*α* are also known to induce ROS generation, which in turn amplifies ROS/TNF*α*-ceramide cycling and exacerbates IR injury [27, 33]. In contrast, in a monoamine oxidase-A deficient animal model the effects of ROS attenuated ceramide generation and IR injury were reduced [26, 34].

 Finally, with administration of SMase inhibitors and SMase knockdown by siRNA, SMase knockout mice have reduced ceramide accumulation during IR and attenuated cell apoptosis and tissue damage through a mechanism that may involve the blockade of C-Jun N-terminal kinase (JNK) activation, the impairment of mitochondrial function, and activation of caspases [21, 22, 35–37]. Taking these data together, ceramide generated from SMases plays a key role in IR-induced later phase damage, and the modulation of ceramide may be an important therapeutic target.

## 3. The De Novo Ceramide Synthesis Pathway and IR

De novo ceramide biosynthesis occurs at the cytosolic side of the endoplasmic reticulum (ER) and mitochondrion and serves as a precursor for the synthesis of more complex sphingolipids, including sphingomyelin and glycosphingolipids, in the Golgi [38, 39]. Ceramide synthases are a family of key enzymes in de novo ceramide synthesis. There are six ceramide synthase isoforms that have been cloned and characterized [40]. Each of the six mammalian ceramide synthases appears to regulate the synthesis of a specific subset of ceramides and displays a unique substrate specificity profile for chain-length and/or saturation of the fatty acid acyl-CoA [41]. Increased ceramide synthesis occurred upon reperfusion in the ischemic area after coronary occlusion in mice, which correlated with the enhanced expression of serine palmitoyltransferase (SPT), the first key enzyme in de novo ceramide synthesis. Myriocin, an inhibitor of SPT, significantly protected the ischemic area from IR injury [37]. Dihydroceramide desaturase, an oxygen sensitive double bond generating enzyme, is the last key enzyme in de novo synthesis. The activity of dihydroceramide desaturase was significantly inhibited and dihydroceramide levels were increased during hypoxia. The elevated dihydrosphingolipids may be involved in exacerbating the IR injury [42]. In a mouse cerebral IR model, after 30 min of middle artery occlusion, followed by 24 hr reperfusion, the content of all ceramide species was elevated without any change in the content of sphingomyelin. Thus, the accumulation of ceramide was consistent with activation of ceramide synthase, rather than the activation of SMases. Moreover, IR-induced stimulation of ceramide synthase activity was very sensitive to the inhibitor fumonisin B1 (FB1) [43]. Studies in cell lines have shown that ceramide generation is involved in the activation of JNK and promotion of Bax translocation to the mitochondria, which also suggested that ceramide may signal through the mitochondrial cell death pathway in response to IR injury [44, 45]. Inhibition of ceramide synthase with both FB1 and JNK3 knockout reduced the accumulation of ceramide and decreased the size of brain infarct regions in a cerebral IR model [43].

## 4. Ceramide Clearance and IR

Interestingly, ceramide concentrations in the myocardium of rats had no apparent change during 30 min of ischemia, but following 3 hours of reperfusion there was a significant elevation. These increases in ceramide were not associated with SMase activity, but rather with reduced ceramidase activity [46]. Furthermore, short periods of anoxia (3 h) followed by reoxygenation (0–5 h) led to a time-dependent increase of caspase activity in human umbilical vein endothelial cells (HUVECs), which was associated with a significant decrease in glucosylceramide synthase mRNA levels and protein expression, but no changes in SMase. After 24 h middle cerebral artery occlusion in rats, increase of ceramide levels also coincided with the decease of glucosylceramide synthase activity in rat brain [47]. These in vivo and in vitro data suggest that the inhibition of ceramide clearance may also contribute to the IR-induced accumulation of ceramide and tissue damage and indicates that strategies to treat IR-induced tissue injury via ceramidase treatment or inhibition of glucosylceramide synthase may also be viable strategies.

## 5. Mitochondrial Damage and IR

Increasing evidence suggests that mitochondria are important intracellular compartments for sphingolipid metabolism, including sphingomyelin and ceramide [48]. Moreover, several enzymes engaged in ceramide metabolism have been identified in mitochondria. With their own set of ceramide synthesizing and hydrolyzing enzymes, mitochondria serve as a specialized compartment of ceramide metabolism in cells. For example, SMases in mitochondria have been identified from zebrafish, mouse, and rat [20, 49, 50]. Purified ceramide synthase from bovine liver mitochondria showed higher activity than that from the ER [51]. Further studies of submitochondrial localization revealed that both outer and inner mitochondrial membranes have enzymatic machinery that can synthesize ceramide [52]. Recent studies demonstrated that ceramide synthase is associated with adenine nucleotide translocase, the inner membrane component of the mitochondrial permeability transition pore (MPTP), and suggested that ceramide generation by ceramide synthase could mediate MPTP activity and mitochondrial Ca2+ homeostasis [53]. An additional source of ceramide in mitochondria is via the reverse activity of neutral ceramidase, and recent reports also describe ceramide formation from acyl-CoA and sphingosine mediated by the coupled activities of mitochondrial neutral ceramidase and thioesterase. Furthermore, mitochondria from neutral ceramidase deficient mice liver exhibited significantly reduced ceramide formation from sphingosine and palmitate, further implicating this “reverse reaction” [52, 54]. Ceramide also can be transported from the ER to mitochondria [55].

Many investigations have also shown a close connection between ceramide signaling and mitochondrial function and that mitochondria are the primary site of ROS production under normal physiologic conditions as well as during ischemia and IR insults [28, 56]. Regardless of the diverse pathways of IR-induced ceramide generation in mitochondria, ceramide-induced apoptosis has common consequences: suppression of the respiratory chain, elevation of ROS formation, discharge of membrane potential, opening of MPTP, and release of proapoptotic proteins [28, 57].

For over a decade multiple studies have shown that mitochondrial dysfunction appears to be one essential step in IR tissue damage, although the impact of ceramide on mitochondrial function during IR is not fully understood and may depend on cell type and stimuli. Indirectly, ceramide activates protein phosphatase 2A (PP2A), resulting in increases of the proapoptotic Bcl-2 family proteins by dephosphorylation of Bax (activation) and Bcl-2 (inactivation) [58]. In addition, ceramide-induced activation of protein phosphatases leads to inactivation of serine/threonine kinase Akt/PKB and activation of proapoptotic Bad [59]. Interestingly, ceramide can trigger Bax into an active conformation and lead to translocation from the cytosol to the mitochondrial membrane with release of cytochrome c during hypoxia/reoxygenation in neuronal cells. Knockdown of SMase or ceramide synthase attenuates Bax translocation [45].

Other indirect mechanisms linking ceramide and mitochondria include ceramide's interaction with protein kinase PKC and mitogen-activated protein kinase (MAPK). Several studies indicate that the increased ceramide levels in heart IR can target PKC *δ*, resulting in activation and mitochondria translocation of PKC *δ* accompanied by cytochrome c release and activation of caspase [60]. Members of the MAPK superfamily, p38 MAPK and JNK, can also be activated by both endogenous ceramide generation in liver IR and addition of exogenous ceramide, followed by translocation to mitochondria, activation/translocation of the Bcl-2 family proteins, initiation of cytochrome c release, and apoptosis [35, 61]. Ceramide can also trigger Ca2+ release from ER to mitochondria. Excessive accumulation of Ca2+ in mitochondria could trigger opening of the MPTP at a high conductance state and lead to cell death [62].

Recent studies have also shown that Sirtuin 3 induces mitochondrial dysfunction by enhancing ceramide biosynthesis via deacylation of ceramide synthase [63]. Ceramide can also suppress the respiratory chain in isolated mitochondria, resulting in increased production of ROS in endothelial cells after hypoxia/reoxygenation. Extensive studies using isolated mitochondria demonstrate that ceramide generation in the outer mitochondrial membrane leads to formation of large pore ceramide-rich rafts, opening of MPTP, and initiation of cytochrome c release [62, 64]. These extensive findings suggest that ceramide generated in both the cytosol and mitochondria may play a critical role in IR-induced mitochondrial injury, dysfunction, and tissue damage. Protection of mitochondrial function via modulation of ceramide could therefore be another essential strategy to prevent IR-induced injury.

## 6. The Protective Effect of Ceramide during Preconditioning

**Figure 2:**
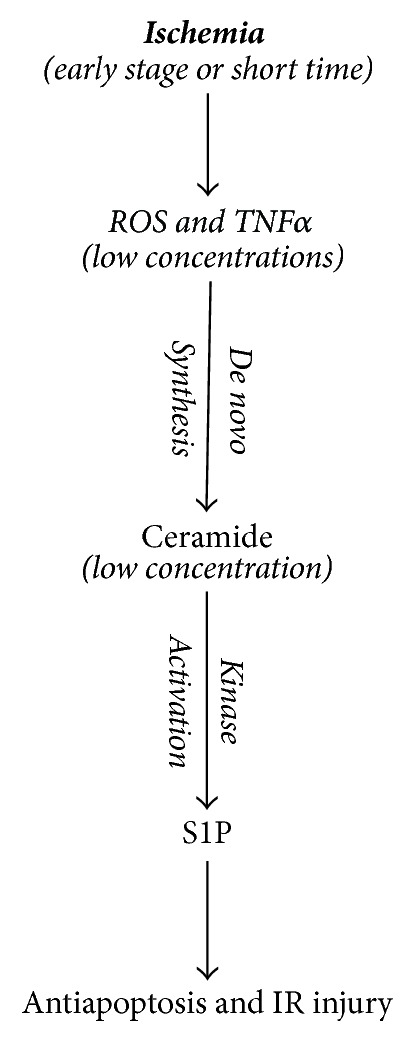
Scheme of ischemic preconditioning and sphingolipids.

Taken together, the information above indicates that ceramide plays a central key in hypoxia/reoxygenation-induced cell death and IR-induced tissue damage. Paradoxically, in recent years several lines of evidence have also demonstrated that ceramide has a protective effect in IR injury when ischemia is used for preconditioning. Ischemic preconditioning (IPC) is a phenomenon whereby brief ischemia provides significant protection against subsequent severe ischemia and reperfusion injury in heart [65, 66], brain [67], and liver [68] (Figure 2).

Little is known about the molecular mechanisms involved in ceramide-induced protection on IR injury. In general, evidence has shown that low concentrations of ceramide promote cell survival while higher concentrations induce cell death [69, 70]. Earlier investigations indicated that IPC promoted a transient accumulation of specific ceramide species, while SM remained unchanged, and treatment with low doses of exogenous ceramide had a similar protective effect on IR insult [65, 71]. Pretreatment with FB1, an inhibitor of ceramide synthase, in rat cortical neurons abolished the neuroprotective effect [71]. These data suggest that de novo ceramide synthesis contributes to IPC-induced ischemia tolerance. Although ROS generation and cytokine activation have been recognized to contribute to IR injury, it has also recently been suggested that ROS and TNF*α* at low doses may also enhance cytoprotective mechanisms [72, 73]. In vivo, IPC promotes ROS and TNF*α* production, and low doses of ROS and TNF*α* can mimic the IPC-induced protective effect in heart and liver [32]. Moreover, the protective effect of TNF*α* is associated with the release of ROS [74]. In addition, an antioxidant, N-2-mercaptopropionyl glycine (MPG), can block both the ROS and TNF*α*-induced cytoprotective effects [75]. The exact roles of ROS and TNF*α* in the cytoprotective effect of ceramide are still elusive. It is suggested that cell survival S1P, derived from increased ceramide hydrolysis, induces cardioprotection via activation of protein kinase C and that ceramide itself may lead to transmembrane receptor clustering and activation of a variety of kinases and phosphatases that regulate the cell antiapoptotic process [76, 77].

S1P is known to be a survival factor for a variety of cell types. A ceramide-S1P rheostat model demonstrated that increases in the concentration of proapoptotic ceramide can be countered by increases in the levels of antiapoptotic S1P [78]. There are two key limiting enzymes in this rheostat, ceramidase and sphingosine kinase (SPHK), regulating the levels of intracellular S1P derived from ceramide. It was reported that exogenous S1P can mimic the preconditioning protective phenotype in an ex vivo rat heart model in which infarct size was significantly reduced [79]. In mouse hearts, SPHK activity was increased following IPC. Further evidence demonstrated that increased intracellular S1P levels induced by IPC resulted from PKC activation and SPHK phosphorylation [80, 81]. Moreover, the cardioprotective effect of S1P could be partially abolished by the sphingosine kinase (N,N-dimethylsphingosine, DMS) or ceramidase (N-oleoylethanolamine, NOE) inhibitors [79, 80], and sphingosine kinase 1 mutation sensitized mouse myocardium to IR injury [82]. Taken together, these studies strongly suggested that the ceramide-S1P rheostat plays a key role in this IPC effect. Strategies to enhance intracellular SIP levels by modulation of ceramidase and/or SPHK activities could be novel and promising therapeutic approaches to prevent tissues from IR injury during severe ischemia and following reperfusion.

The majority of S1P is stored in platelets and erythrocytes. It is secreted by an essential transporter [83] and plays numerous biological roles, such as antiapoptotic process and proliferation. However, a growing body of evidence has shown S1P can be pathogenic in various diseases [84]. Increased S1P levels can enhance inflammation and trigger the S1P-ceramide recycling pathway, causing apoptosis and tissue damage as well [85]. The pathogenic effect of S1P should be also considered in IR injury.

In summary, ceramide accumulation has been demonstrated in various models of IR and has been implicated as an important mediator of apoptosis in the injured tissue. Ceramide accumulation during IR could occur through a combination of mechanisms, and the effects may range from protective to damaging based on timelines, the extent of ceramide production, tissue type, and/or extent of ischemia and reperfusion. The IR-induced accumulation of ceramide appears to be a general phenomenon for many organs, including heart, kidney, liver, brain, and intestine, but with differences. Each of these organ systems will be discussed below.

## 7. Heart

Heart damage caused by IR is well recognized as a significant cause of morbidity and mortality (thrombolysis, myocardial infarction, cardiac surgery, primary percutaneous coronary intervention, etc.). The first study demonstrating that hypoxia/reoxgenation induced a progressive accumulation of ceramide was in cardiomyocytes [15] where in the rat heart left coronary occlusion model the ceramide content in ischemic myocardium was increased to 155% in early ischemia and further elevated to 250% after 3 h reperfusion. Further in vitro and in vivo findings suggested that ceramide was involved in cardiomyocyte apoptosis and IR-induced heart injury, although the mechanisms of IR-induced accumulation of ceramide are incompletely understood. Several lines of evidence indicate that sphingomyelin hydrolysis by SMases is responsible for the ceramide elevation in cardiac IR injury [25, 65, 66]. Pharmacological inhibitors of SMases, such as D609, amitriptyline, and desipramine, as well as the expression of domain-negative FAN, prevented IR-induced accumulation of ceramide and attenuated cardiomyocyte apoptosis and IR heart injury [20, 77, 86]. An interesting study in IR-induced mouse heart injury revealed ceramide elevation and inhibition of SPHK, which led to upregulation of the ceramide/S1P ratio resulting in cardiac tissue damage [34]. In addition of SMase activation, enhanced de novo ceramide synthesis also may be involved in IR heart injury in mice [37]. Another report showed that accumulation of ceramide in reperfused rat heart was associated with reduced ceramidase activity, rather than enhanced SMase activity [46]. Thus, multiple mechanisms appear to be involved.

There is also strong evidence demonstrating that IPC enables cardiomyocytes and heart to become more resistant to subsequent severe ischemia and reperfusion [65, 66], and it is known that ROS and antioxidant play key roles in the effect of IPC on ceramide-induced cardioprotection. Pretreatment with antioxidants prevented ceramide generation and abrogated this effect [33, 74]. In addition, the IPC-induced cardioprotective effect may also result from PKC activation and SPHK activation to enhance intracellular S1P levels in rat heart [80]. This effect of S1P could be blocked by a sphingosine kinase inhibitor and by using a sphingosine kinase 1 mutant mouse model [82].

## 8. Brain

Stroke is a major cause of long-term disability. Ischemic stroke occurs when cerebral arteries are occluded or stenosed by emboli or local atherosclerotic disease. Restoration of blood flow following ischemic stroke can be achieved by means of thrombolysis or recanalization. However, in some clinical cases, reperfusion may exacerbate the injury initially caused by ischemia, producing a cerebral reperfusion injury. The signaling cascades activated by cerebral ischemia and IR that may promote neuronal death are not well understood.

Kubota et al. provided the first evidence that ceramide was increased in an ischemic human brain resulting from an acute case of cerebral occlusion [87]. Ceramide accumulation has been subsequently reported in multiple models of neural ischemia, including cerebral cortex and hippocampus ischemia, and in models of IR, including rat cerebral and coronary artery occlusions [19, 36, 88, 89]. There are diverse molecular mechanisms involved in ceramide-induced cerebral ischemia and IR injury, and it appears that the pathway of ceramide activation depends on the severity of the insult to the brain.

In severe cerebral ischemia and IR, ceramide accumulation resulted from activation of acid SMase and inhibition of glucosylceramide synthase [19, 47]. It is also known that integrin-associated Lyn kinase suppressed acid SMase activity, promoting cell survival, and after 1 h of middle cerebral artery occlusion and 48 h of reperfusion in the mouse brain the disruption of the intergrin-Lyn kinase complex led to activation of acid SMase and accumulation of ceramide [90]. Consistent with these results, severe ischemia-induced brain injury is decreased in acid SMase knockdown mice and following treatment of healthy mice with an acid SMase inhibitor [36].

In mild ischemia and IR, the de novo ceramide synthesis pathway also can contribute to ceramide accumulation [71, 89]. In a mouse cerebral IR model, de novo ceramide generation appeared to promote cell death by abrogating the mitochondrial respiratory chain [63], as inhibition of ceramide synthesis with FB1 reduced the size of cerebral infarct regions [43]. However, this model of ceramide action is complicated by the neuroprotective effects observed in IPC. Brief ischemia protects primary cultured neurons from hypoxia-induced cell death; ceramide levels were elevated while sphingomyelin remained unchanged following brief hypoxia. Treatment with exogenous, low dose ceramide had a similar neuroprotective effect [71]. This protective effect was abolished by pretreatment with FB1 [89]. Together, these later data suggest that the de novo ceramide synthesis pathway, rather than sphingomyelin/ceramide pathway, is involved in two stages: neuroprotection during brief ischemia and cerebral injury in mild ischemia and IR.

## 9. Liver

Hepatic IR damage, which can occur in diverse settings including liver resection and transplantation, trauma, hemorrhagic shock, or liver surgery, is a serious clinical complication that may compromise liver function because of extensive hepatocellular loss. Initial evidence regarding the role of ceramide showed that it was elevated in rat liver after cold ischemia and warm reperfusion during transplantation [14]. In the total rat liver ischemia/reperfusion model, both neutral and acid SMase activities were initially decreased during the early phase of ischemia, and acid SMase activity was increased during the later phase (over 1 h). The initial inhibition of SMase activities may contribute to the enhanced S1P levels due to a negative crosstalk between S1P and acid SMase [91, 92]. Accumulation of TNF*α* and activation of SMase also were observed during reperfusion of the ischemic lobe of rat livers [30]. TNF*α* gene knockout mice also exhibited hepatoprotection against IR-induced liver injury [32]. In a murine model of warm hepatic IR injury, an early phase of ceramide elevation was observed at 30 min after IR due to activation of acid SMase and inhibition of sphingomyelin synthase, followed by acid ceramidase stimulation. The later phase of ceramide elevation occurred at 6 h after IR due to only activation of acid SMase and unchanged acid ceramidase activity. Administration of SMase inhibitors decreased ceramide accumulation during hepatic IR and attenuated cell necrosis, cytochrome c release, and caspase activation [21]. In contrast, administration of a ceramidase inhibitor enhanced ceramide generation and exacerbated hepatic IR injury [35]. These results suggest that ceramide generated from TNF*α* mediated activation of acid SMase, irrespective of acid ceramidase, plays an important role in IR-induced liver damage and that the modulation of ceramide levels by inhibition of SMase and/or activation of ceramidase may be of therapeutic relevance.

## 10. Kidney

Ischemia/reperfusion injury is an unavoidable complication after kidney transplantation and is associated with delayed graft function and graft rejection. The first report implicating ceramide in renal IR injury was published by Zager et al. in 1997 [16]. In this whole mouse renal IR model, ceramide levels in kidney showed transient reduction after 45 min of ischemia, followed by a 2- to 3-fold increase during the reperfusion phase [93]. Of interest, the decrease of ceramide paralleled a decline of both acid and neutral SMase activities during the ischemia phase, but the increase during reperfusion was not associated with increased SMase activities, which in fact decreased further [23]. This SMase suppression may be accounted for by ceramide driven S1P production, resulting in inhibition of SMase activity [91, 92]. Using an in vivo model of unilateral renal occlusion, the renal injury was attenuated by inhibiting de novo ceramide synthesis with FB1, but not by suppressing SMase activity with the D609 inhibitor [94]. In the normal mouse renal cortex, C24, C22, and C16 ceramide are the main species, constituting 70%, 10%, and 20% of the total ceramide content, respectively. C16 ceramide was significantly increased, and all others mildly increased, after IR. Interestingly, IR induced an apparent shift towards unsaturated (versus saturated) fatty acids within the C22 and C24, but not the C16 ceramide pool [93]. These findings suggested that de novo ceramide biosynthesis plays a key role in IR-induced C16 ceramide accumulation and renal injury.

## 11. Intestine

Intestinal IR injury can occur in diverse conditions, including small bowel transplantation, acute mesenteric ischemia, hemorrhagic, and traumatic or septic shock [95]. IR injury is a major difficulty in small bowel transplantation. It is known that intestinal IR leads to severe destruction of distant tissues because of damage to the intestinal mucosal barrier, which causes a systemic inflammatory reaction and multiple organ dysfunctions [96]. A few reports have demonstrated that apoptosis was a major mode of rat and mouse small intestinal epithelial cell death induced by intestinal IR [97, 98]. The first study suggesting a role for ceramide in intestinal IR injury was published by Liu et al. in 2007 [99]. In the rat IR model of clamping superior mesenteric artery, intestinal IR caused intestinal mucosal epithelial apoptosis and accumulation of ceramide, followed by upregulation of SMase mRNA expression [100]. In the small rat bowel graft model following cold ischemia and subsequent warm reperfusion, an elevation of ceramide and extensive apoptosis were observed in the intestinal tissue [101]. To determine whether de novo ceramide synthesis was involved in intestinal IR injury, a rat splanchnic artery occlusion and reperfusion model was implemented. By clamping both the superior mesenteric artery and the celiac artery for 45 min followed by reperfusion, all rats died during a 4 h reperfusion period due to significant accumulation of ceramide, elevated production of TNF*α*, and extensive apoptosis in the intestine. Pretreatment with FB1 dramatically reduced these proapoptotic reactions [102]. These findings reveal that ceramide generated by SMase and do novo biosynthesis both contribute to the development of intestinal IR injury.

## 12. Summary

**Figure 3:**
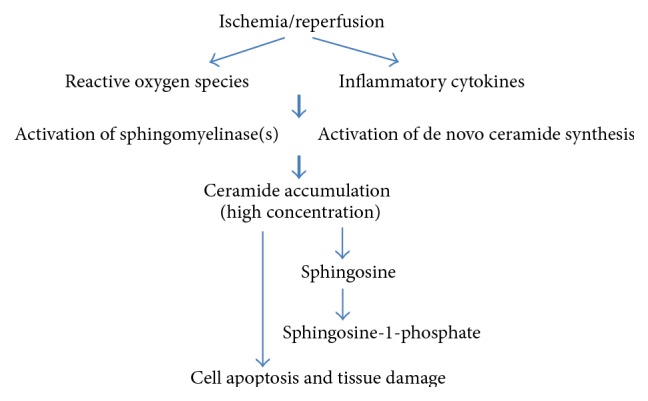
Flowchart of IR injury and sphingolipids.

During ischemia and reperfusion, the generation of excess ROS and inflammatory cytokines in IR tissue is the initial key response. These oxidative and inflammatory stress stimuli activate two major ceramide generation pathways (sphingomyelin/ceramide and de novo synthesis), resulting in significant ceramide accumulation, cell apoptosis, and tissue damage (Figure 3). Regardless of the diverse pathways and complicated mechanisms underlying ceramide generation, it is widely accepted that the generation of ceramide is central to the pathogenesis in ischemia/reperfusion injury. Generally, the severity of IR-induced tissue injury depends on the phase and extent of ischemia and reperfusion, which in turn is associated with the amount and selective pathway of ceramide generation. Tissues also generate protective S1P, likely by activation of ceramidase and/or SPHK, during very early phases of ischemia, which may cause transient reduction of ceramide and S1P-induced inhibition of SMase. Following a brief ischemia, increased ROS generation induces small amounts of ceramide by activation of the de novo synthesis pathway. During this early phase of ischemia, ceramide generation is generally protective in nature. After prolonged ischemia following reperfusion, the massive production of ROS and TNF*α* results in significant accumulation of ceramide via activation of the sphingomyelin/ceramide signaling pathway, combined with alterations of downstream ceramide metabolism (e.g., glycosylceramide synthase, ceramidase, and SPHK), which in turn engages downstream pathways of inflammation and apoptosis contributing to mild to severe IR injuries. Administration of pharmacological inhibitors of SMase and ceramide synthase, genetic knockdown by siRNA, and use of SMase knockout mice reduced ceramide accumulation during IR and attenuated cell apoptosis and tissue damage. It is well known that mitochondria are a major site of ROS generation and have their own set of ceramide generating enzymes. Ceramide-induced mitochondrial dysfunction appears to be an essential step in IR-induced tissue damage. Although ceramide contributes to the development of IR-induced tissue injury, it presents differently between organs. The sphingomyelin/ceramide signaling pathway is dominant for ceramide-induced IR injury in heart, liver, brain, and intestine, whereas ceramide accumulation primarily results from the de novo biosynthesis pathway in IR-injured kidney. Further studies are required to further understand the role of ceramide in ischemic organs and IR injury, but based on extensive data accumulated over the past two decades it is clear that strategies that reduce ceramide by modulations of ceramidase and/or SMase activities may represent novel and promising therapeutic approaches to prevent or treat IR injury in diverse clinical settings.
